# Gender difference in the associations between health literacy and problematic mobile phone use in Chinese middle school students

**DOI:** 10.1186/s12889-023-15049-4

**Published:** 2023-01-20

**Authors:** Dan-Lin Li, Sizhe Wang, Daoxu Zhang, Rong Yang, Jie Hu, Yanni Xue, Xuexue Huang, Yuhui Wan, Chen-Wei Pan, Jun Fang, Shichen Zhang

**Affiliations:** 1 School of Public Health, Medical College of Soochow University, 199 Ren Ai Road, Suzhou, 215123 Jiangsu Province China; 2 MOE Key Laboratory of Population Health Across Life Cycle/ Anhui Provincial Key Laboratory of Population Health and Aristogenics, 81th Meishan Road, Hefei, 230032 Anhui Province China; 3 Department of Social Medicine and Health Education, School of Public Health, Nanjing Medical University, 101 Longmian Road, Nanjing, 211166 Jiangsu Province China; 4 State Key Laboratory of Bioreactor Engineering, Shanghai Frontiers Science Center of Optogenetic Techniques for Cell Metabolism, Frontiers Science Center for Materiobiology and Dynamic Chemistry, Shanghai Key Laboratory of New Drug Design, School of Pharmacy, East China University of Science and Technology, 130 MeiLong Road, Shanghai, 200237 China; 5 Department of Epidemiology, School of Public Health, Cheeloo College of Medicine, Shandong University, 44th Wenhuaxi Road, Jinan, 250000 Shandong Province China; 6 Department of Maternal, Child and Adolescent Health, School of Public Health, Tongji Medical College, Huazhong University of Science and Technology, 13 Hangkong Road, Wuhan, 430030 Hubei Province China; 7 Department of Maternal, Child and Adolescent Health, School of Public Health, Anhui Medical University, 81th Meishan Road, Hefei, 230032 Anhui Province China; 8 School of Public Health and Health Management, Anhui Medical College, 632 Furong Road, Hefei, 230601 Anhui Province China; 9 Faculty of Pharmaceutical Science, Sojo University, Ikeda 4-22-1, Kumamoto, 860-0082 Japan

**Keywords:** Health literacy, Addictive behavior, Adolescent, Gender difference, China

## Abstract

**Background:**

Problematic mobile phone use (PMPU) is becoming increasingly popular and has serious harmful effects on physical and mental health among adolescents. Inadequate health literacy (HL) is related to some risky behaviors and mental health problems in adolescents. Nevertheless, few studies have explored the relationship between HL and PMPU and the gender difference in the relationship among Chinese adolescents. The aim of this study was to examine the associations between HL and PMPU and explore gender difference in the associations.

**Methods:**

A total of 22,628 junior and senior high school students (10,990 males and 11,638 females) in 6 regions of China participated in this study. HL and PMPU were measured by self-report validated questionnaires. Chi-square tests and logistic regression analysis were conducted in the study.

**Results:**

Logistic regression analysis showed that students with inadequate HL are likely to have PMPU (*OR* = 2.013, 95% *CI*: 1.840–2.202), and different degrees of association can be seen in six dimensions. Besides, in both males and females, students with inadequate HL had a higher risk of PMPU (*OR*_male_ = 1.607, 95% *CI*: 1.428–1.807; *OR*_female_ = 2.602, 95% *CI*: 2.261–2.994). Regarding the gender difference, the results showed that males had more PMPU than females, and the difference was more significant for students with adequate HL than those with inadequate HL (*OR*_inadequate_ = 1.085, 95% *CI*: 1.016–1.159; *OR*_adequate_ = 1.770, 95% *CI*: 1.490–2.101). Similarly, there were associations in the six dimensions.

**Conclusions:**

HL decreases PMPU, and males have a higher risk of PMPU than females. These findings suggest a reasonable strategy to reduce PMPU by improving the HL level of adolescents.

**Supplementary Information:**

The online version contains supplementary material available at 10.1186/s12889-023-15049-4.

## Background

In recent years, the usage of mobile phones has increased dramatically. A report from the WHO estimated approximately 6.9 billion mobile phone users worldwide in 2014 [1]. In developed countries such as the United States, approximately 64% of the population used smartphones in 2017 [2]. In addition, smartphone usage in developing countries, such as India, is also expected to reach 36.0% by 2018, and approximately 50.6% of Turkish teenagers are problem phone users [3, 4]. Meanwhile, the China Youth Internet Behavior Survey Report of 2015 indicated that the number of Chinese youth internet users reached 287 million, accounting for 85.3% of the total Chinese youth population, which is much higher than the overall national internet users in 2015 (50.3%) [5]. Obviously, mobile phone use is very widespread among teenagers around the world.

Mobile phones not only bring us convenience but also harm our health. Studies have revealed that excessive use of mobile phones can trigger various physical disorders, such as blurred vision, local pain and obesity [6, 7]. Moreover, the use of smartphones may cause some addictive behaviors and further induce mental health problems, including phone addiction, sleep disorders, anxiety and depression [8–10]. Problematic mobile phone use (PMPU) refers to “Failure to regulate personal cell phone use that may have a negative impact on daily life” [11]. Because of the high sensitivity of adolescents to PMPU, concerns have been raised about the possible influences on health of mobile phone use, particularly on children and adolescents [8]. It is thus important to develop strategies to countervail PMPU in adolescents. Some strategies have been used in many families for example limiting the use of cellphone by specific software or implementation plan, which are proven effective. Besides, it has been reported that exercise intervention, improvement of self-control, or psychological treatment that are strongly associated with better health literacy (HL), are effective to improve PMPU [11]. It is thus reasonable to reduce PMPU by improving HL.

HL is defined as how well a person can obtain and understand health information and services, and use them to make good health decisions [12]. Additionally, Nutbeam proposed that HL is a more advanced cognitive and literacy skill that can be used to actively participate in everyday activities and apply new information to changing circumstances [13]. More notably, the theoretical framework of adolescence HL suggests that HL can have different degrees of influence on a variety of health behaviors [14]. Many studies have examined and demonstrated the negative association between HL and health-risk behaviors, such as smoking, alcohol use, self-harm, screen time, and suicidal behaviors [15–17], which strongly supports the important role of HL in adolescent health promotion [18].

Adolescence is an important period in everyone’s life, and improving health status during this period is thus of vital importance and can greatly affect people’s lifelong health [19]. However, adolescents often lack correct health awareness and health management ability, so they frequently fail to make correct health choices, which will lead to a series of health risk behaviors, such as sexual risk behavior, and internet addiction [20, 21]. Regarding internet addiction, some literatures have pointed to that there is a difference in mobile phone use between males and females, and male have a greater possibility of having PMPU and internet addiction [22, 23]. However, controversy exists in this issue, and some other studies have shown that females are more addicted to phones than males [13, 24]. It can be seen that the gender difference in mobile phone use is an issue worthy of discussion.

Nevertheless, most of the previous studies were based on relatively small populations, and few Chinese studies concerned the relationship between HL and PMPU as well as the gender difference in the relationship. In this study, we investigated the association of HL and PMPU in Chinese adolescents and tackled the gender difference in the association based on a questionnaire survey among junior and senior high school students in six cities of China to provide guidance for reducing PMPU in Chinese adolescents.

## Material and methods

### Study participants

From November 2015 to January 2016, convenience sampling was used to select samples from junior and senior high schools in 6 cities in China, including urban and rural areas. The 6 cities are Xinxiang(a city in northern Henan Province), Shenyang (the capital of Liaoning Province), Bengbu(a city in northeastern Anhui Province), Chongqing (one of China’s four direct controlled municipalities), Ulanqab(a city in the central Inner Mongolia Autonomous Region) and Yangjiang(a city in the southwest coast of Guangdong Province). Then, we selected 8 schools from each city, and 4–6 classes were selected for each grade of every school for investigation.

### Questionnaire data

The questionnaires consist of questions on demographic variables (i.e., gender, grade, registered residence, accommodation type, type of school, household structure, parents’ educational level, self-reported family economy and number of friends), the Self-rating Questionnaire for Adolescent Problematic Mobile Phone Use (SQAPMPU) and the Chinese Adolescent Interactive Health Literacy Questionnaire (CAIHLQ), as described below.

The CAIHLQ was used to assess the HL level. It consists of 31 items grouped into 6 domains, including physical activities (e.g., ‘Following a planned exercise program’), interpersonal relationships (e.g., ‘Taking times with your family or friends’), stress management (e.g., ‘Balance time between study and play’), self-actualization (e.g., ‘Feeling each day is very meaningful’), health awareness (e.g., ‘Constricting sugars and food containing sugar’), and dietary behavior (e.g., ‘Eating 200–400 g of fresh fruit each day’) [25]. Each item is rated on a 5-point Likert scale (never and no desire, never but with desire, occasionally and irregularly, often, and routinely), and the total score ranges from 31 to 155, with higher scores indicating better HL [25]. Participants in this study were categorized as adequate HL groups when their scores were ≥ 120. In this study, the internal consistency test showed that the Cronbach’s α coefficient was 0.910 and 0.662 to 0.847 for the six subscales, and the reliability and validity of the CAIHLQ have been demonstrated in previous studies [15, 26].

The SQAPMPU includes 13 items and 3 domains, including withdrawal symptoms (e.g., ‘If I don’t have a phone, I will feel overwhelmed’); craving (e.g., ‘I always feel that I don’t have enough time to use my phone’), and psychosomatic effects (e.g., ‘Too much mobile phone use leads to insufficient sleep’) [27]. Each item is responded to on a 5-point Likert scale (never, occasionally, sometimes, often, and always). Exploratory factor analysis showed that the cumulative variance contribution rate of the questionnaire was 59.13%, and Cronbach’s α coefficient was 0.87. In this study, the Cronbach’s α coefficient was 0.923. The Cronbach’s α coefficients of the three dimensions are 0.879 (withdrawal symptoms), 0.711(craving), and 0.832(psychosomatic effects). According to previous studies, students in this study were categorized as PMPU when this score was ≥ *P*_75_ [28].

### Procedure

The study was conducted in accordance with the Declaration of Helsinki and obtained approval from the Ethics Committee of Anhui Medical University (March 1, 2014; Approval No. 20140087). Informed consent was obtained from all subjects and their parents. In addition, we trained investigators through lectures, discussions and practice. During the investigation, the investigators explained the purpose of the investigation and the instructions for completing the questionnaire, and then each participant completed a self-report questionnaire within 20 to 30 minutes. The investigators withdrew the questionnaires at the scene. After the investigation, the investigators sorted out and recorded the questionnaires.

### Statistical analysis

Statistical analysis was performed by SPSS ver. 23.0 for Windows (SPSS, Inc., Chicago, IL). Cronbach’s alpha analysis was performed to determine the reliability of the survey. The chi-square test was used to compare the prevalence of PMPU among different demographic variables. Binary logistic regression models were performed to examine the association between the 6 domains of HL and PMPU in all students, males, and females. In addition, subgroup analysis was used to separate gender differences in different groups of HL. Analyses were adjusted based on control for key demographic and socioeconomic variables. Statistical significance was set at *P* < 0.05.

## Results

### Univariate analysis

After excluding 507 invalid questionnaires (missing rate ≥ 5%), a total of 22,628 questionnaires were included in the survey (effective rate was 97.8%); 10,990 were male (48.6%), and 11,638 were female (51.4%). Participants had a mean age of 15.18 years (SD = 1.79), and the overall CAIHLQ mean score for all students was 104.06 ± 18.68. The CAIHLQ scores were normally distributed, and the variability of the data was consistent. Table 1 presents the prevalence of PMPU by frequency characteristics. Male students had a significantly higher prevalence of PMPU than female students [male (26.5%) vs. female (24.4%)]. Likewise, the prevalence of PMPU in senior high school students, only children, key school students, students without friends and students with poor family economic conditions was significantly higher than that in matched groups, e.g., junior high school students, more than one child, nonkey school students and so on (*P* < 0.05 for each). However, there were no statistically significant differences in registered residence and parents’ educational levels (*P* > 0.05 for each, Table 1).

**Table 1:** Frequency characteristics of problematic mobile phone use (PMPU) in Chinese adolescents

Variable	***n*** (***%***)	PMPU		***χ***^***2***^
		No (***n*** = 16,876)	Yes (***n*** = 5752)	
Gender				13.972^***^
Male	10,990 (48.6)	8074 (73.5)	2916 (26.5)	
Female	11,638 (51.4)	8802 (75.6)	2836 (24.4)	
Grade				74.736^***^
Junior high school	11,993 (53.0)	9227 (76.9)	2766 (23.1)	
Senior high school	10,635 (47.0)	7649 (71.9)	2986 (28.1)	
Registered residence				0.967
Rural	10,882 (48.1)	8148 (74.9)	2734 (25.1)	
Urban	11,746 (51.9)	8728 (74.3)	3018 (25.7)	
Accommodation type				43.292^***^
Boarding student	11,320 (50.0)	8227 (72.7)	3093 (27.3)	
Commuting student	11,308 (50.0)	8649 (76.5)	2659 (23.5)	
Type of school				9.395^**^
Key school	11,588 (51.2)	8542 (73.7)	3046 (26.3)	
Non-key school	11,040 (48.8)	8334 (75.5)	2706 (24.5)	
Household structure				6.118^*^
Only child	9720 (43.0)	7169 (73.8)	2551 (26.2)	
More than one child	12,908 (57.0)	9707 (75.2)	3201 (24.8)	
Father’s education level ^*a*^				0.001
< High school degree	13,006 (58.0)	9707 (74.6)	3299 (25.4)	
≥ High school degree	9424 (42.0)	7035 (74.6)	2389 (25.4)	
Mother’s education level ^*b*^				0.072
< High school degree	14,335 (63.9)	10,693 (74.6)	3642 (25.4)	
≥ High school degree	8105 (36.1)	6059 (74.8)	2046 (25.2)	
Self-reported family economy				68.203^***^
Bad	3240 (14.3)	2244 (69.3)	996 (30.7)	
General	16,345 (72.2)	12,411 (75.9)	3934 (24.1)	
Good	3043 (13.4)	2221 (73.0)	822 (27.0)	
Number of friends				56.084^***^
None	599 (2.6)	379 (63.3)	220 (36.7)	
Few	14,535 (64.2)	10,768 (74.1)	3767 (25.9)	
More	7494 (33.1)	5729 (76.4)	1765 (23.6)	

Statistical methods: Chi-square test; ^*a*^198 students have no father, ^*b*^188 students have no mother. *PMPU* Problematic mobile phone use. ^***^ *P* < 0.001; ^**^ *P* < 0.01; ^*^ *P* < 0.05

### Logistic regression analysis

After adjusting for the effect of gender, grade, accommodation type, type of school, household structure, self-reported family economy, and number of friends, inadequate HL was significantly associated with an increased risk of PMPU (*OR* = 2.013, 95% *CI*: 1.840–2.202). Meanwhile, inadequate HL in six domains was significantly positively correlated with PMPU (Table 2).

**Table 2:** Associations of HL and PMPU among junior and senior high school students

Variables	PMPU		
	***n*** (%)	Crude ***OR***(95%***CI***)	Adjusted ***OR*** (95%***CI***)^**a**^
HL			
Inadequate	5069 (27.8)	2.085 (1.910–2.276)^***^	2.013 (1.840–2.202)^***^
Adequate	683 (15.6)	1.000	1.000
Physical activities			
Inadequate	4690 (26.4)	1.282 (1.189–1.383)^***^	1.211 (1.120–1.308)^***^
Adequate	1062 (21.9)	1.000	1.000
Interpersonal relationships			
Inadequate	4594 (28.2)	1.754 (1.631–1.885)^***^	1.713 (1.591–1.844)^***^
Adequate	1158 (18.3)	1.000	1.000
Stress management			
Inadequate	4903 (27.5)	1.762 (1.625–1.911)^***^	1.690 (1.556–1.835)^***^
Adequate	849 (17.7)	1.000	1.000
Self-actualization			
Inadequate	4905 (27.2)	1.636 (1.508–1.775)^***^	1.583 (1.457–1.719)^***^
Adequate	847 (18.6)	1.000	1.000
Health awareness			
Inadequate	4882 (27.5)	1.735 (1.601–1.880)^***^	1.704 (1.571–1.848)^***^
Adequate	870 (17.9)	1.000	1.000
Dietary behavior			
Inadequate	4602 (26.9)	1.394 (1.266–1.500)^***^	1.318 (1.251–1.453)^***^
Adequate	1150 (20.9)	1.000	1.000

*OR* Odds ratio, *CI* Confidence interval, *HL* Health literacy, *PMPU* Problematic mobile phone use

^a^Adjusted for gender, grade, accommodation type, type of school, household structure, self-reported family economy, and number of friends

^***^ *P* < 0.001

### Gender difference in the association between HL and PMPU

**Fig. 1:**
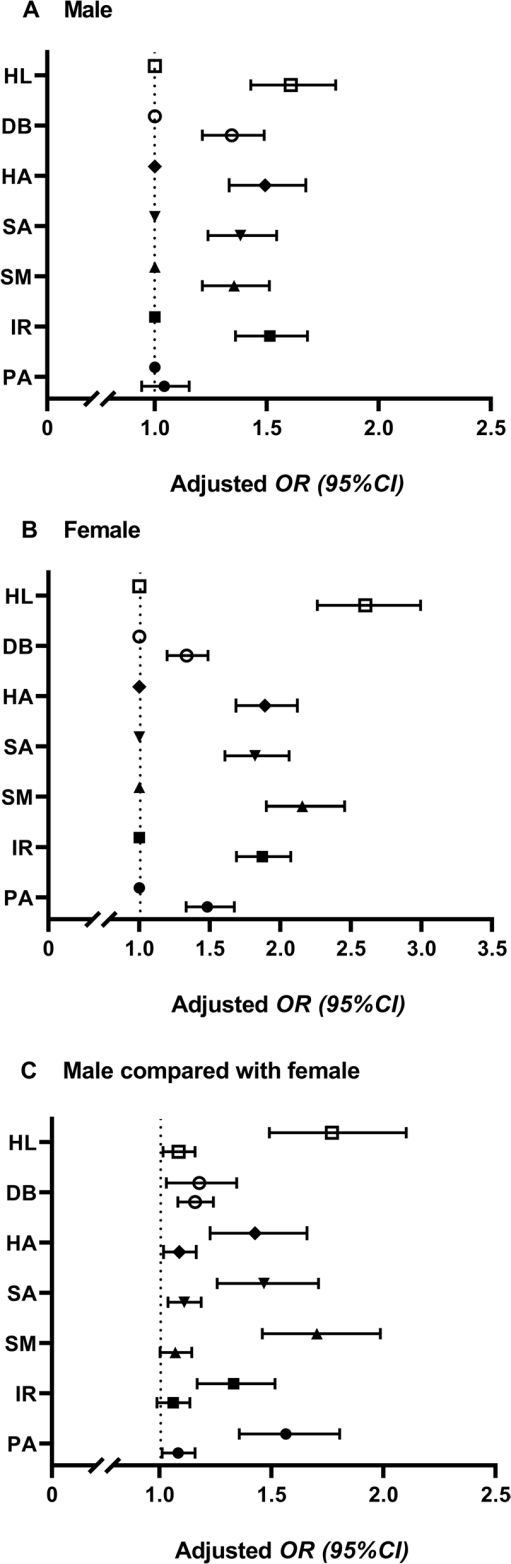
Association of HL with PMPU in male (**A**) and female (**B**), and the gender comparison (**C**). *OR*, odds ratio; *CI*, confidence interval; HL, health literacy; PMPU, problematic mobile phone use; PA, physical activity; IR, interpersonal relationships; SM, stress management; SA, self-actualization; HA, health awareness; DB, dietary behavior. The data were adjusted for grade, accommodation type, type of school, household structure, self-reported family economy, and number of friends. ● PA; ■ IR; ▲ SM; ▼ SA; ◆ HA; ○ DB; □ HL

As shown in Fig. 1, students with inadequate HL had a high risk of PMPU in both males (*OR*_male_=1.607, 95% *C*I: 1.428–1.807) and females (*OR*_female_=2.602, 95% *CI*: 2.262–2.994). This relationship was also seen in the six dimensions of HL with PMPU (Fig. 1A, B, Table A1). Besides, regarding gender, the results showed that males had a higher risk of PMPU than females in all the students, regardless of adequate or inadequate HL (*OR*_inadequate_=1.085, 95% *CI*: 1.016–1.159; *OR*_adequate_=1.770, 95% *CI*: 1.490–2.101). This association could also be seen in six dimensions in students with adequate interpersonal relationship HL (Fig. 1C, Table A1).

## Discussion

In this study, we examined the association between PMPU and HL in junior and senior high school students in China. As hypothesized, students with inadequate HL had more PMPU than those with adequate HL. In addition, males had a higher risk of PMPU than females among students with both inadequate and adequate HL.

The results revealed that males, senior high school students, boarding students, key school students, students with lower family income and no friends had a higher prevalence of PMPU than the matched groups, which is consistent with previous studies [29–32]. According to previous studies, friendships play an important role in problematic behaviors among adolescents [29]. When adolescents lack the companionship of friends, they relieve loneliness by keeping in touch with their peers through online dating, thus leading to PMPU, which is consistent with our study that the students with fewer friends have more PMPU [33, 34]. Additionally, the prevalence of PMPU in commuting students was lower, which may be related to the stricter supervision of parents when students are at home and parents could accompany them and give them support more [35]. However, our results indicated that parents’ educational levels have no significant effects on PMPU among adolescents, whereas a previous study reported the opposite results [36]. The different results may be related to the choice of the population and the inconsistency of the measurement tools, which warrants further investigation in the future.

Our findings suggested that adolescents with inadequate HL (both HL and six domains) are more likely to have PMPU. This may be because adolescents with inadequate HL cannot read, understand, and obtain information sufficiently, resulting in the inability to fully benefit from media interventions, events or educational projects [37]. As known, health awareness is a vital indicator of a person’s awareness of health problems, and individuals unaware of their health problems may be more prone to aggravated health risk behaviors, such as PMPU [38]. Studies have shown that the problems of internet addiction arose among adolescents due to the lack of health awareness and emotional management ability [39, 40]. As the Billieus PMPU access model demonstrates, emotional management and interpersonal relationship are important ways to lead to PMPU. Others studies have also pointed out that good interpersonal relationships can indirectly reduce adolescents’ PMPU by alleviating loneliness and motivation because loneliness can lead to excessive and compulsive use of mobile phones to relieve symptoms and deal with bad emotions, leading to a vicious cycle and thus increasing the risk of PMPU [41]. These findings are in accordance with our study, which suggested that high emotional pressure can lead to problematic mobile use [42]. Besides, stress and stress management are negative predictors of mobile phone addiction [40, 43]. Moreover, a study on adolescents showed that dietary behavior was linked with stress [44], and stress may affect PMPU in an indirect way through unhealthy dietary behavior. In addition, the relation between physical activity and PMPU can be explained by the use of time, because more physical activity will naturally reduce the time spent on mobile phones. Taken together, it is reasonable to utilize HL and the multiple dimensions of HL for the prediction of adolescents’ PMPU.

Furthermore, we found that PMPU was more likely to occur in males, which was inconsistent with a study in Saudi Arabia [45], and it may result from males usually showing extroverted personality, which makes them more active in trying new technologies, such as the desire to possess the mobile phone model, to play games or assess the internet, while females use mobile phones mostly for social contact, and fulfilling their need for closeness, communication and emotional expressions [46]. Moreover, males’ impulsive personality may make them more likely to engage in risky health behaviors even with adequate health awareness [47]. Interestingly, the present study revealed that among students with adequate HL, the gender difference in PMPU was slightly greater than that among students with inadequate HL. Based on these results, we considered that inadequate HL can weaken the differences between males and females in PMPU. Nevertheless, the mechanisms by which HL affects this difference remain to be further studied.

This study was an epidemiologic study with large samples, and we selected participants from both rural and urban regions, considering the difference in the different socioeconomic conditions. In addition, the SQAPMPU and CAIHLQ were developed on based the characteristics of Chinese teenagers and have excellent reliability, constructive validity and pertinence. However, some limitations should also be noted. First, only six cities were included, and the validity of this study for students in other regions is not fully clear and needs further investigation. The second issue is the reliance on the self-report nature of the data, in which recall and reporting biases could not be avoided. Finally, the cross-sectional design cannot fully reflect the causal relationships. Longitudinal studies are needed in the future to clarify the causal relationships between HL and PMPU.

## Conclusion

In summary, our results suggested a negative association between HL and PMPU. Meanwhile, males have a higher risk of PMPU than females, and students with adequate HL have a slightly higher gender difference in PMPU than students with inadequate HL. From a prevention standpoint, in order to reduce the prevalence of PMPU among adolescents, it should be considered to improve adolescents’ HL levels, especially for males, by, for example, carrying out health education classes or lectures regularly in school and by subscribing to health knowledge.

## Supplementary Information

**Additional file 1: Table A1.** Odds ratio (95% *CI*) associated with HL and PMPU in male and female, and the gender comparison.

## Data Availability

The datasets generated and/or analysed during the current study are not publicly available, but are available from the corresponding author on reasonable request.
