# Supplementary material for: Gender difference in the associations between health literacy and problematic mobile phone use in Chinese middle school students
Source: BMC Public Health. 2023 Jan 20;23:142. doi: 10.1186/s12889-023-15049-4 (PMC9854151; doi:10.1186/s12889-023-15049-4)
Supplement: Supplementary file 1 — Additional file 1: Table A1. Odds ratio (95% CI) associated with HL and PMPU in male and female, and the gender comparison. [file 12889_2023_15049_MOESM1_ESM.docx]

**Table A1** Odds ratio (95% *CI*) associated with HL and PMPU in male and female, and the gender comparison.

| **Variables** | | **Male** | | **Female** | | **Male compared with female** |
| --- | --- | --- | --- | --- | --- | --- |
|  | Crude *OR* (95%*CI*)^a^ | Adjusted *OR* (95%*CI*)^a^ | Crude *OR* (95%*CI)*^a^ | Adjusted *OR* (95%*CI*)^a^ | Adjusted *OR* (95%*CI*)^a^ |  |
| HL | |  |  |  |  |  |
| Inadequate | | 1.705 (1.520-1.913)^***^ | 1.607 (1.428-1.807)^***^ | 2.730 (2.377-3.316)^***^ | 2.602 (2.261-2.994)^***^ | 1.085 (1.016-1.159)^*^ |
| Adequate | | 1.000 | 1.000 | 1.000 | 1.000 | 1.770 (1.490-2.101)^***^ |
| Physical activity | |  |  |  |  |  |
| Inadequate | | 1.107 (1.002-1.2235)^*^ | 1.042 (0.941-1.153) | 1.603 (1.423-1.806)^***^ | 1.481 (1.331-1.673)^***^ | 1.083 (1.012-1.159)^*^ |
| Adequate | | 1.000 | 1.000 | 1.000 | 1.000 | 1.564 (1.356-1.804)^***^ |
| Interpersonal relationships | |  |  |  |  |  |
| Inadequate | | 1.572 (1.417-1.744)^***^ | 1.513 (1.360-1.682)^***^ | 1.916 (1.732-2.119)^***^ | 1.871 (1.688-2.073)^***^ | 1.061 (0.989-1.136) |
| Adequate | | 1.000 | 1.000 | 1.000 | 1.000 | 1.331 (1.168-1.516)^***^ |
| Stress management | |  |  |  |  |  |
| Inadequate | | 1.439 (1.292-1.604)^***^ | 1.354 (1.212-1.511)^***^ | 2.265 (1.999-2.565)^***^ | 2.155 (1.900-2.445)^***^ | 1.070 (1.002-1.144)^*^ |
| Adequate | | 1.000 | 1.000 | 1.000 | 1.000 | 1.702 (1.459-1.986)^***^ |
| Self-actualization | |  |  |  |  |  |
| Inadequate | | 1.454 (1.304-1.622)^***^ | 1.382 (1.237-1.544)^***^ | 1.907 (1.686-2.158)^***^ | 1.819 (1.606-2.061)^***^ | 1.110 (1.038-1.186)^**^ |
| Adequate | | 1.000 | 1.000 | 1.000 | 1.000 | 1.466 (1.257-1.710)^***^ |
| Health awareness | |  |  |  |  |  |
| Inadequate | | 1.551 (1.385-1.737)^***^ | 1.493 (1.332-1.674)^***^ | 1.922 (1.724-2.154)^***^ | 1.890 (1.685-2.120)^***^ | 1.088 (1.018-1.164)^*^ |
| Adequate | | 1.000 | 1.000 | 1.000 | 1.000 | 1.426 (1.226-1.658)^***^ |
| Dietary behavior | |  |  |  |  |  |
| Inadequate | | 1.412 (1.277-1.562)^***^ | 1.343 (1.212-1.488)^***^ | 1.396 (1.254-1.554)^***^ | 1.334 (1.196-1.487)^***^ | 1.159 (1.082-1.241)^***^ |
| Adequate | | 1.000 | 1.000 | 1.000 | 1.000 | 1.177 (1.030-1.344)^*^ |

*OR*, odds ratio; *CI*, confidence interval. HL, health literacy; PMPU, problematic mobile phone use. ^a^ Adjusted for grade, accommodation type, type of school, household structure, self-reported family economy, and number of friends. ^***^ *P* < 0.001; ^**^ *P* < 0.01; ^*^ *P* < 0.05
